# Medical Students' Perception of Automated Note Feedback After Simulated Encounters

**DOI:** 10.1111/tct.70273

**Published:** 2025-11-17

**Authors:** Saurabh K. Bansal, Manajyoti Yadav, Jianing Zhou, Rebecca A. Ebert‐Allen, Ryan M. Klute, William F. Bond, Suma Bhat

**Affiliations:** ^1^ Department of Internal Medicine University of Illinois College of Medicine at Peoria Peoria Illinois USA; ^2^ Department of Computer Science University of Illinois Urbana‐Champaign Illinois USA; ^3^ Jump Simulation, an OSF HealthCare and University of Illinois College of Medicine at Peoria Collaboration Peoria Illinois USA; ^4^ Department of Emergency Medicine University of Illinois College of Medicine at Peoria Peoria Illinois USA; ^5^ Department of Electrical and Computer Engineering University of Illinois Urbana‐Champaign Illinois USA

**Keywords:** automated feedback, medical student, model notes, natural language processing (NLP)

## Abstract

**Background:**

Grading medical student patient notes (PNs) is resource‐intensive. Natural language processing (NLP) offers a promising solution to automatically grade PNs. We deployed an automated grading system that uses NLP and explored the perceived value of PN feedback.

**Approach:**

The automated system graded written notes after two standardized patient encounters by third‐year medical students. The system generated an individualized report on ‘items found’ and ‘items not found’ in the history, physical examination, and diagnosis sections, which was shared with students for feedback via a web‐based interface. By rotation, block students received either the automated case feedback first or the faculty‐written model note feedback first (the pre‐intervention baseline).

**Evaluation:**

After reviewing feedback, students completed surveys for both automated feedback and model note feedback and participated in follow‐up focus groups. In total, 44 students received feedback, 37 completed surveys, and 28 participated in focus groups. Qualitative themes that emerged suggested the automated feedback was visually appealing and allowed for easy comparison of items found vs. missing, which would help improve students' documentation skills. Model note appeared trustworthy.

**Implications:**

We found automated systems can be a potential tool for formative feedback on note writing activity although in terms of quality it does not surpass the pre‐existing feedback methods, such as model note feedback used in our study. Order effects may have influenced these perceptions and the small sample size limits generalizability. Tested software had occasional errors in recognizing a phrase or showing a false positive.

## 1 Background

Credible, timely feedback after direct observation is crucial for improving learner performance. It is most effective when it reinforces strengths and identifies omissions [1]. Grading of written notes, whether done formatively or summatively, provides valuable feedback to the learners. In our experience patient notes (PNs) after standardized patient (SP) encounters are graded manually which is a time‐consuming process. Due to limited faculty availability, it often does not occur or if it does then it happens in a delayed fashion. This creates a missed opportunity to give feedback to learners.

Hansen et al [2] in a scoping review revealed a paucity of literature on note‐writing feedback. Literature review noted that using checklists or rubrics allows standardization of assessment but still requires manual faculty input [3]. On the other hand Yudkowsky et al. [4] successfully showed that non‐faculty raters can grade a note using checklists and scoring systems. Despite that, locally and globally, training of non‐faculty raters by faculty, and the time delay to conduct grading, remain a challenge. Researchers have shown that automated systems can complement existing feedback methods to improve feedback quality, quantity, timeliness and consistency [5]. We introduced a home‐grown automated grading system that uses natural language processing (NLP) methods to give feedback to the students on their written notes.

*We introduced a home‐grown automated grading system that uses natural language processing (NLP) methods to give feedback to the students on their written notes*.

While automated note grading is not a novel concept, its use in providing formative feedback to students is new. Additionally, studying the perception of students on the quality of the feedback has not been previously reported. Other attempts that do exist on automated PN grading are rare and have focused mainly on high‐stakes examinations and proprietary systems [6].

Structure of our research aligns with the educational framework of deliberate practice proposed by Ericsson. Medical students are developing new skills of note writing and when timely and informative feedback is given after task completion, it will lead to skill improvement upon subsequent practice [4].

In this study our students conducted interviews of SPs and wrote progress notes. An automated system was used to give them feedback on PN. Our goal was to explore the perceived value of PN feedback to the medical students using qualitative and survey methods. Given the paucity of literature on PN feedback in general, we sought feedback on both the automated and the pre‐innovation model note PN feedback methods. This allowed us to benchmark the perceived value of the current feedback method and determine whether the automated feedback was perceived to have similar or greater value to the students.

*Goal was to explore the perceived value of PN feedback to the medical students using qualitative and survey methods*.

## 2 Approach

### 2.1 Participants and Setting

From 2022–2023, MS3s at the end of 8‐week Internal Medicine (IM) clerkship at a regional campus of a major U.S. medical school completed SP simulations. Demographics such as age, gender or race were not collected from the respondents. However, the study participants represent a reflective sample of a U.S. medical school, comprising individuals from a variety of backgrounds and diversely represented. Forty‐four students participated in the activity. Consent to participate was obtained prior to exercise and this effort was approved by the Peoria Institutional Review Board under expedited review. Students who chose not to participate in the study were still offered the full educational experience of simulation and feedback. As part of the curriculum, learners interacted with the SPs for two simulated cases, one headache and one back pain on the same day. They were provided 20 min to gather information and 10 min to type their notes into a Qualtrics (Qualtrics, Provo, UT, USA) form that included sections for history, physical, differential diagnosis and its justification, and suggested work‐up.

*Learners interacted with the SPs for two simulated cases, one headache and one back pain on the same day*.

### 2.2 Intervention and Patient Note Feedback Processes

**FIGURE 1:**
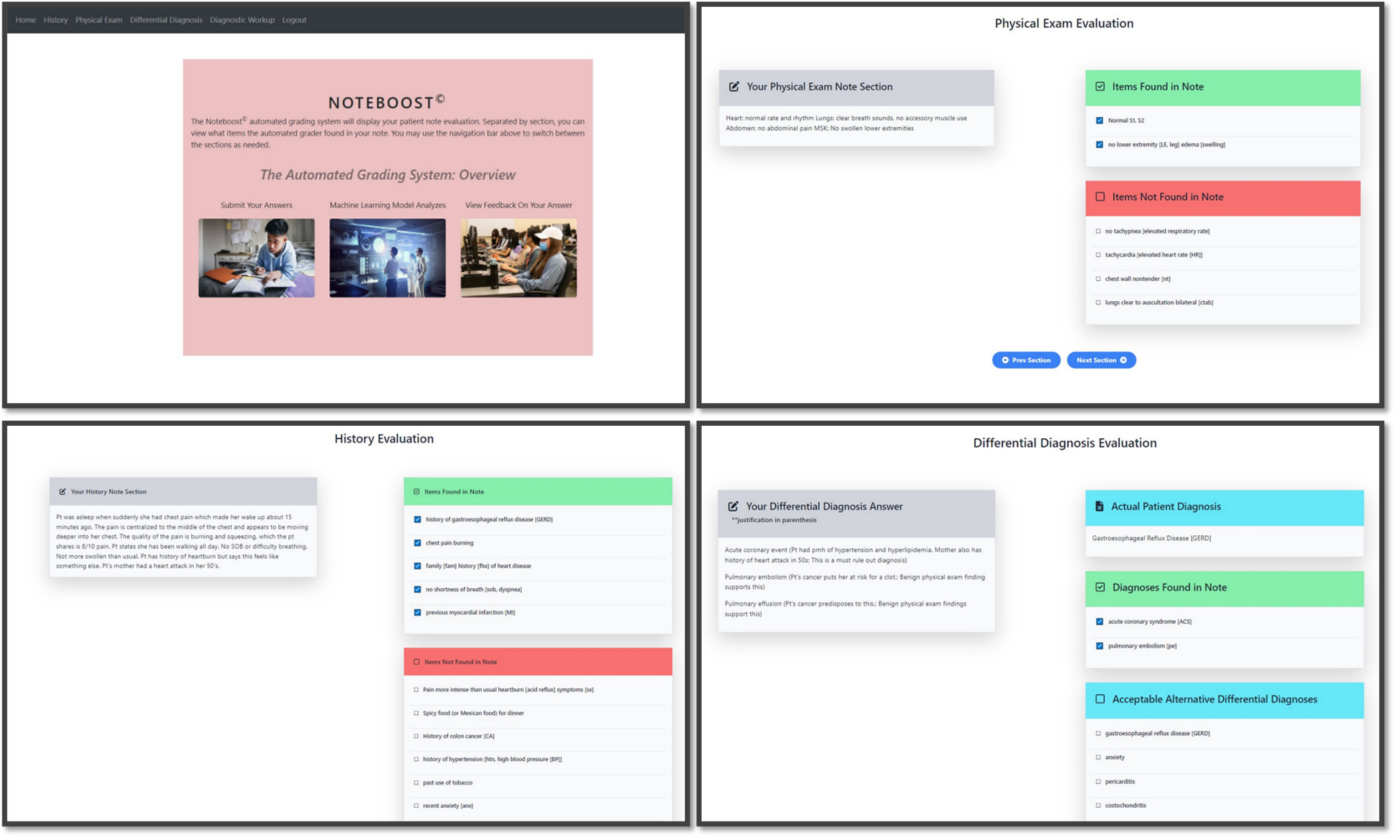
NoteBoost automated grading interface.

The automated feedback was given via the internally developed NoteBoost software system [7]. This system uses NLP methods to find the checklist items in a patient note and checks for phrases and paraphrases that are close matches to a checklist item. The feedback from this system appears as a list of ‘items found’ and ‘items not found’ in the history and physical section. Additionally, it shared the case diagnosis, alternative diagnoses, and whether the student found those diagnoses. An example image of the feedback interface is shown in Figure 1. Details regarding automated system integration and its reliability verification check are placed separately in Data S1.

The prior study condition was the use of faculty‐generated model notes for feedback. For self‐feedback, students received a printed copy of their own written note, and a pre‐written case‐specific model faculty note for comparison. Case‐specific model notes were the baseline method of self‐feedback in the clerkship prior to this study. They were pre‐written by faculty (authors MY and SKB).

## 3 Evaluation

### 3.1 Design

This was a mixed‐methods evaluation design that collected focus group data for qualitative analysis and survey data for quantitative analysis. Both sets of data were treated equally. Thematic analysis was performed on the qualitative dataset. To get final results, we used a convergent design and triangulated both sets of data at the conclusion of the study [8].

**FIGURE 2:**
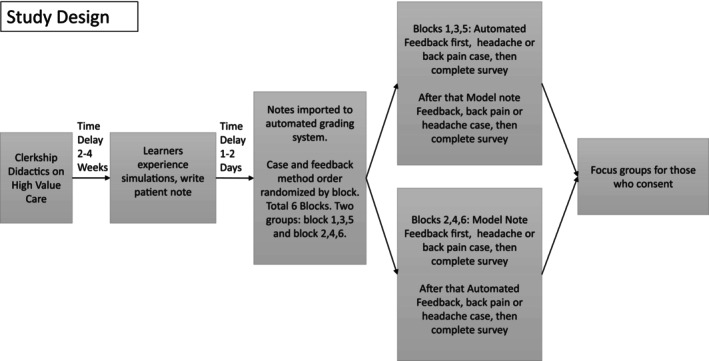
Study design.

Students received both types of feedback on both cases the same day. We recognized that the sequence in which learners receive feedback can influence their perception of its usefulness. To minimize this order effect, the case assigned to receive automated feedback first versus model note feedback first was alternated through clerkship blocks. For example, in one block, students received automated feedback on the back pain case first, followed by model note feedback on the headache case, while in the next block, the sequence and case‐feedback pairing were reversed. Study design implements principles of deliberate practice allowing improvement driven by feedback. Clerkship directors, authors SKB and MY, were not physically present during consenting, survey administration, or focus group sessions to avoid any undue influence on the students. The study design is available in Figure 2.

### 3.2 Data Collection

#### 3.2.1 Focus Groups

Students were invited to participate in a semi‐structured focus group after completing both feedback surveys. Focus groups were led by investigators REA and RK who have no grading or authority roles over the students, and each lasted approximately 30 min. Each group had 7–10 students. Focus groups allowed consensus development between the group participants as well as interaction of divergent views [9]. Focus group questions were designed to elicit the merits and limitations of each feedback system independently, without prompting students to rank or directly compare the two approaches. The focus group leaders were allowed to ask follow‐on questions such as ‘tell me more’ or ‘can you please clarify.’ The full list of questions is available in Data S2.

#### 3.2.2 Data Collection—Surveys

The survey domains included influence on perception of performance, feedback clarity, and perceived accuracy among other things. The survey was developed by faculty consensus, and pilot tested with pre‐research participants to ensure clarity. The survey questionnaire is provided for reference in Data S3. All responses were collected on a 6‐point Likert scale of *Strongly disagree*, *Disagree*, *Somewhat Disagree*, *Somewhat Agree*, *Agree* and *Strongly agree*. The survey also included a retrospective pre‐post self‐efficacy question [10] on a 10‐point scale phrased as follows: ‘My ability to write a patient note for this chief complaint BEFORE and AFTER the simulation case feedback.’

#### 3.2.3 Data Analysis—Qualitative

For qualitative data, we performed thematic analysis using an inductive approach. The inductive approach is data‐driven, and themes are informed by the data itself and are not reflective of researchers' own interests or the beliefs of the subjects [11]. We used MaxQDA (2023, Verbi Software, Berlin, Germany) software for qualitative data which automatically maintains audit trails and all changes and edits were visible to the investigators. Anonymized transcripts were prepared and proofread by R.K. and R.E.A. After the first two groups were completed, investigators M.Y. and S.K.B. analyzed the data individually and generated initial codes by reviewing every comment. Once all comments were coded, subthemes were generated and grouped into broader themes. Thereafter, they met compared results with each other, reconciled differences and refined or merged themes where appropriate. Subsequently, investigators W.B. and R.E.A. reviewed the thematic structure and further refined it, reorganizing themes and subthemes based on conceptual commonalities to produce a consolidated framework. Furthermore, consensus meetings were conducted between W.B., S.K.B., M.Y. and R.E.A. and adjustments were made to ensure the wording of themes and subthemes is closely reflective of assigned comments and the thoughts expressed in those comments match the assigned themes. To explore certain areas in greater depth and find newer themes we modified questions for the last three groups. Using the existing thematic structure W.B., M.Y. and S.K.B., reviewed the additional three transcripts with the ability to add new themes. It was followed by the same process of consensus meetings to ensure themes' accuracy and accurate matching of comments with the themes. Data collection concluded after the fifth focus group when thematic saturation was reached.

#### 3.2.4 Data Analysis—Quantitative

Survey analysis included basic descriptive statistics. Data were analyzed using Microsoft Excel (Microsoft, Redmond, Washington). Given the small sample size, the model note was not intended to be a true comparison arm but rather to better understand the strengths and weaknesses of the pre‐study condition. The evaluation was not designed or powered to assess superiority–inferiority.

#### 3.2.5 Evaluation Results

A total of 44 students completed the activity and received feedback during the study period. Out of those, 37 completed the post‐activity evaluation survey, and 28 participated in five separate focus groups with each group ranging from 7 to 10 students.

#### 3.2.6 Qualitative Results

The formative nature of this exercise encouraged robust participation. The activity occurred at the end of an 8‐week IM clerkship; therefore, students appeared comfortable comprehending and evaluating the quality of the feedback. The maturation effect was evident, as the students during the second half of the academic year offered more elaborate and in‐depth responses. Qualitative analysis revealed several themes and sub‐themes and correlating comments that are illustrated in Table 1. Highlighting some of the prominent themes and subthemes that emerged from the analysis here: students found the automated feedback visually appealing, with an example quote stating that ‘it was easy to go through the whole thing broken down into different components’. Automated feedback has higher learning and retention value, stating that ‘it makes us think rigidly in terms of checklist for the differentials’. Students commented that automated feedback can be used in conjunction with the faculty feedback to complement each other. Students reported that the automated system had occasionally erred in phrase recognition or awarded false positives where credit for an item found was given when it should not have.

**TABLE 1:** Themes, subthemes and pertinent quotations from student focus group sessions.

Themes and subthemes	Quotations exemplar quotes
**Theme 1:** Automated feedback was clear, easy for comparison and helped impress pertinent findings **Subthemes** Feedback is clearer, visually appealing and easy for comparison. Automated grading helps impress pertinent findings. It will help improve note writing skills.	‘It was easy to like, go through it just clicking next and seeing the whole thing broken down into different components.’ ‘A lot of things that I had missed that I saw on the automated feedback was pertinent negatives. And I think that's something I miss generally too, so going forward continuing to kind of include pertinent and others into the history.’ ‘It was very neutral. It's not like “you missed this, bad medical student.”’ ‘The organizational skills … It helps problem‐based assessments at some point. It helps you just naturally get into the habit of thinking about it in that structure.’
**Theme 2:** Automated feedback enhances learning because it was more direct, had higher retention value, and reinforced clinical reasoning **Subthemes** Point wise display and direct feedback in automated grading has better learning value. It improves clinical reasoning skills. Automated grading could be useful to complement faculty feedback.	‘At least that's just a checklist, the automated one, but it's a very thorough checklist and so it's easier to look because it tells you what you missed.’ ‘It helps with thinking more like a rigid manner, in terms of … a checklist for differentials.’ ‘We get personalized feedback from preceptors who are assigned to us. I do not think this should in any way replace that … But I do think that the automated process could be an addition to that process and students can easily see like what they missed and what they included.’
**Theme 3:** Automated feedback method includes phrase recognition limitations and abstract nature of feedback **Subthemes** Automated feedback does not explain thought process on why a particular finding is more important than others It showed false positives (gave credit for ‘item found’ when it should have not). It had phrase recognizing limitation.	‘If it was added like the sensitivity specificity of like different physical exams and what it would indicate or what the sensitivity specificity of performing that physical exam, what it would tell you, that would be very cool.’ ‘I found a couple of things that they had thought I had written in my note, but I had not actually written them. So, it's just like a false positive.’ ‘My note said the pain does not radiate, but then I got marked for not saying that because the explicit term was ‘does not radiate to the legs.’
**Theme 4:** Model note feedback is beneficial in showing good example, but feedback is harder for comparison **Subthemes** Model note can be trusted in showing a good example. Model notes help with organization and phrasing. Model notes are more realisticModel note explains the differential and plan. Model note feedback sets rigid expectations. Model note feedback is harder for comparison.	‘There is a little bit of, like trust that I have too in the standard or in the like model note thing. Because I know there was a physician who knew this case, theoretically did the exam themselves. ‘ ‘I think organization. Like the flow of how you are writing this story so that how to phrase certain things that you might not be so sure how to do that.’ ‘The model notes are the real thing and more practical in that way.’ ‘The model note explained why they have the differential, which was like one of the other great part of the model note.’ ‘A little bit harder to read, because I'm kind of reading line by line and then reading through my note and then reading through their note, reading my note, just back and forth. It's kind of annoying, you know?’
**Theme 5:** More note feedback desired, perhaps iterative, in pre‐clinical years **Subthemes** Earlier feedback could help set future expectations for notes. Automated grading could be useful in pre‐clinical years. Feedback should be sequential/iterative process.	‘If we are in a clinical setting, you know, if we had a model note like before, if our preceptor or Dr. provided us a model note on the first day, I feel like our notes would be much better.’ ‘Might be useful in M1 and M2 where you do get more time, immediately have learned all this information, so you really try to do that structured note, and include all the details, it might be very helpful for them.’

*Automated feedback has higher learning and retention value*.

Students also found model note feedback beneficial and felt it can be trusted as a good example, a quote stating, ‘there is a bit of trust because I know a physician knowing this case wrote it.’ Although they found model note harder to compare because of the need to sift through the entire note and self‐identify individual pieces of information that were missing.

#### 3.2.7 Quantitative Results

**FIGURE 3:**
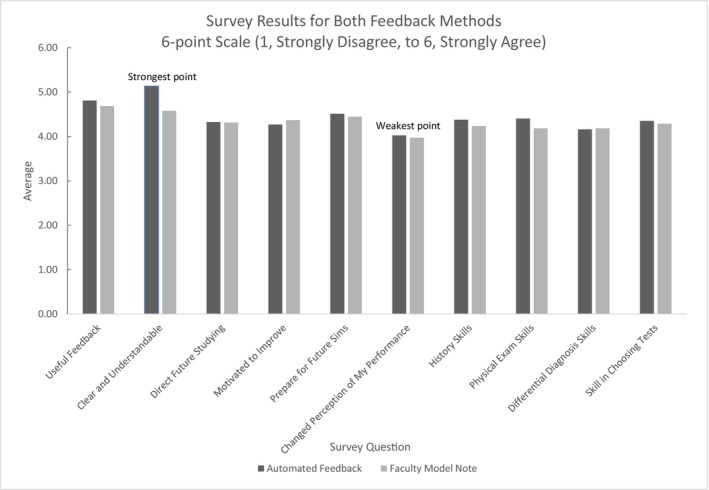
Student responses to 6‐point Likert scale survey questions, *n* = 37 for automated feedback, *n* = 38 for faculty model note. The full survey questions are available in Data S3.

Out of 44 third‐year medical students, 37 completed post‐activity surveys. The survey questionnaire is provided in Data S3. The post‐event evaluation revealed improvement in average retrospective pre‐post self‐efficacy in note writing with the automated feedback, increasing from 70% (SD = 0.16) to 78.5% (SD = 0.16) (t = −3.81, *p* < 0.001). Descriptive survey results are available in Figure 3. Survey results for automated feedback in areas including clarity, perceived utility, and impact on skill building in history taking and physical examination, were marginally positive but no statistical differences were found across feedback methods.

*Improvement in average retrospective pre‐post self‐efficacy in note writing with the automated feedback, increasing from 70% (SD = 0.16) to 78.5% (SD = 0.16) (t = −3.81, p < 0.001)*.

## 4 Implications

There exists a formative feedback gap on PN writing [2] and a scarcity of methods recommended to build note writing skills [2, 10]. Our effort demonstrated an automated system as an adequate method to provide PN feedback. Students perceived the feedback from the automated system as having advantages in terms of clarity, directness, and ease of comparison. Colour‐coded format from the automated system gave them clear visual cues on the presence or absence of an item in a ‘yes’ or ‘no’ format. The feedback highlighted key features and pertinent negatives in a checklist format which students liked and felt it would enhance their clinical reasoning and differential diagnosis generation. The neutral tone of the feedback made it constructive and easier to absorb and appeared to be free from faculty bias. Despite the occasional occurrence of false positives (credit given when not due) generated by the automated system, students consistently reported the feedback as valuable. This can be attributed to the fact that key elements remained clear, and it still provided the message students needed to know. Had this been a high‐stakes assessment, learners would likely have contested for any points they felt were inappropriately missed.

*Feedback from the automated system is having advantages in terms of clarity, directness, and ease of comparison … the neutral tone of the feedback made it constructive and easier to absorb and appeared to be free from faculty bias*.

Survey results demonstrated that students found both automated and model note feedback meaningful for improving note writing skills. In focus groups, students mentioned that they found model notes trustworthy knowing that they were written by the faculty. Although, students found it harder to compare because of the attention it required to parse through what was missing. Another disadvantage of self‐comparison feedback methods such as model notes is that faculty is not aware of what was missed, so it lacks the rigorousness of grading, and it is not clear whether students had identified everything that was missing from their notes. Our findings indicate that, for the intended purpose of formative feedback both approaches offer complementary strengths. From the student's perspective, they found both methods useful, differing only slightly across survey items. We feel this reflects that students have an inherent tendency to improve their performance and better themselves and they view feedback as an opportunity for growth. Ericsson's framework echoes this phenomenon, highlighting that goal‐oriented practice when combined with immediate feedback leads to the development of expertise.

*Students mentioned that they found model notes trustworthy knowing that they were written by the faculty*.

Upon literature review on these methods, we noted authors Spadafore et al. [12] have used NLP technology to assess the quality of narrative feedback and assist grading in written medical examinations [7, 12, 13], Cianciolo et al. used it to score written diagnostic justifications on simulation cases [13], and Schaye et al. used it to classify the quality of clinical reasoning from admission notes written in the clinical environment [14]. These studies assessed whether feedback produced by NLP systems met the quality standards from a faculty perspective. In contrast, our study used NLP technology to assess the perspective of end users, namely the students. By setting this up, it takes NLP technology use to the next level and closer to integration into educational practice.

*It takes NLP technology use to the next level and closer to integration into educational practice*.

During focus group sessions, multiple students commented that iterative feedback will be advantageous, a point that corresponds to the core foundation of deliberate practice. Based on our experience, we think the use of automated feedback will make such an approach more manageable for faculty and may prove more organized and useful for students. To extend it further, it can be expected that the time faculty would have spent on note grading may now be focused on analyzing gaps in learner performance and supporting the remediation of low performers with actionable feedback [15].

In terms of limitations, our study was focused on early application and took place at a single institution with a small sample size, which limits generalizability and order effect may have influenced the perceptions. The timing delay in feedback was due to the absence of an end‐to‐end student privacy compliant system for note capture, as well as the need for accuracy checks. This will be resolved as the technology improves, after which grading speed depends on computational resources applied. For the calculation of survey results, ranked choice responses may introduce slight imprecision in the interpretation of the means. Additionally, the automated grading system used in this study does not address writing style or explain the likelihood ratio of a particular physical exam finding or has an occasional error in phrase recognition. All these findings indicate the continued importance of faculty input. However, with the rapidly evolving scope of NLP‐based software, along with the application of large language models such as ChatGPT, many of these shortcomings will likely be overcome in the near future [16, 17].

In conclusion, we found through our study that automated systems can be a potential tool for formative feedback on note writing activity based on students' perception, although in terms of quality it does not surpass the pre‐existing feedback methods, such as model note feedback used in our study.

*Automated systems can be a potential tool for formative feedback on note writing activity*.

## Ethics Statement

This project was approved under expedited review by the Peoria Institutional Review Board, Protocol Number 1871746.

## Conflicts of Interest

At the time of study execution, Drs. Bhat and Bond did not have a conflict of interest. Drs. Bhat and Bond are currently affiliated with Sapient.AI Corporation, an educational technology company. Sapient.AI did not provide any software or services for this project.

## Supporting information


**Data S1:** Additional information about automated grading system.

**Data S2:** Focus group questions.

**Data S3:** Learner feedback survey.

## Data Availability

The data that support the findings of this study are available from the corresponding author upon reasonable request.
