# Supplementary material for: Medical Students' Perception of Automated Note Feedback After Simulated Encounters
Source: Clin Teach. 2025 Nov 17;22(6):e70273. doi: 10.1111/tct.70273 (PMC12624243; doi:10.1111/tct.70273)
Supplement: Supplementary file 1 — Data S1: Additional information about automated grading system. [file TCT-22-e70273-s001.docx]

**Additional Information about Automated Grading System**

**Automated system implementation**

NoteBoost automated system has achieved high levels of accuracy and inter-rater reliability when compared with faculty raters.^7^ Case specific checklist items for grading were manually entered into the system, which took only a brief time (minutes to hours) after faculty consensus on the items. The base grading system was refined on a small sample of pilot notes for each case used in this study, where faculty did phrase-level annotation to expand phrases that lead to credit. Learner notes were anonymized and captured in Qualtrics, then exported to NoteBoost. Students received feedback 2-3 days later via NoteBoost interface. At this stage of development, there was a need to export the PNs into NoteBoost, and to conduct checks to ensure the system was functioning accurately, that is why it took 2-3 days to get feedback ready for delivery.

**Automated System Reliability Verification Check**

The accuracy of the automated system NoteBoost was captured and reported separately predating this study.^7^ To check reliability for this effort, a random sample of five notes (approximately 10% of the notes available) each from back pain and headache cases were manually graded by authors MY and SKB using identical checklists and then correlated with the grades from the automated system using Cohen’s kappa. Faculty-machine agreement for the back pain case (kappa of 0.64 (SE = .06) and 0.66 (SE = 0.06)), and for the headache case (kappa of 0.81 (SE = 0.04) and 0.77 (SE = 0.05)) were above the generally accepted level of 0.6 deemed acceptable for formative feedback.^13^

Our assessment is that from a technology deployment standpoint, checklists are easier to translate for automated grading. Our automated software provided good faculty-machine agreement for cases with kappas above 0.6. While below the typical bar of 0.8 for high stakes assessment,^13^ they are acceptable for feedback intended for improvement. For now, NLP based technologies can be safely used for low stakes or formative assessments.
