# Supplementary material for: Medical Students' Perception of Automated Note Feedback After Simulated Encounters
Source: Clin Teach. 2025 Nov 17;22(6):e70273. doi: 10.1111/tct.70273 (PMC12624243; doi:10.1111/tct.70273)
Supplement: Supplementary file 2 — Data S2: Focus group questions. [file TCT-22-e70273-s002.docx]

**Focus Group Questionnaire**

1. Please share your experience with the quality of feedback you have received on your patient notes in the past and today.
2. What did you find most useful about the automated grading and feedback on your patient notes?
3. What, if any, limitations or difficulties did you experience with the automated feedback method?
4. What skills, if any, does the note writing and feedback process help develop?
5. What were the most useful aspects of self-feedback using comparison to a good model note?
6. What, if any, limitations or difficulties did you find with the good model note comparison process?
7. What skills, if any, does the note writing and model note comparison feedback process help develop?
8. How did the feedback systems change your perception of your performance?
9. Are there ways that the feedback methods will impact your patient care notes in future?

Based on results from early focus groups, the questions were edited to better capture students’

opinions specific to the automated feedback system:

1. Please describe your preferred methods or ideal features for receiving note feedback.
2. How will the feedback from the automated note feedback system help you write notes going forward?
3. Besides helping with note writing, what other skills, if any, does the automated note feedback activity help you develop?
4. How did the note-writing and automated feedback activity help you develop skill in interpreting or prioritizing history or physical exam findings?
5. How did the note-writing and automated feedback activity help you develop skills in building a differential diagnosis?
6. Where else in your medical school training could you see the automated note feedback system usefully applied?
7. What, if any, limitations or difficulties did you experience with the automated feedback method?
8. How else do you feel the automated note feedback system could be improved?
9. Did any of the feedback systems change your perception of your performance? If it did, how? If not, why not?
10. Looking ahead in future, what barriers or challenges, if any, do you perceive in implementing today’s feedback into your patient notes?
