# Supplementary material for: Medical Students' Perception of Automated Note Feedback After Simulated Encounters
Source: Clin Teach. 2025 Nov 17;22(6):e70273. doi: 10.1111/tct.70273 (PMC12624243; doi:10.1111/tct.70273)
Supplement: Supplementary file 3 — Data S3: Learner feedback survey. [file TCT-22-e70273-s003.docx]

**Survey on Feedback Quality after completion of Simulation activity**

We value your opinion of the quality of the feedback shared with you about your performance as a learner throughout the simulation experience. Your responses on this survey are anonymous and will not in any way be

tied to your performance or grades.

This survey is completely voluntary.  You may choose not to participate or stop at any time.

Thank you for helping us improve feedback to learners from simulation experiences.

Regarding your experience with the **AUTOMATED FEEDBACK OR [GOOD MODEL NOTE]:**

Which case did you receive automated or [good model note] feedback for?

- Headache case
- Back pain case

The automated grader or [good model note] provided useful feedback on my performance.

- Strongly disagree
- Disagree
- Somewhat disagree
- Somewhat agree
- Agree
- Strongly agree

The automated grader or [good model note] provided feedback that was clear and understandable.

- Strongly disagree
- Disagree
- Somewhat disagree
- Somewhat agree
- Agree
- Strongly agree

The automated or [good model note] case feedback will help direct my future studying.

- Strongly disagree
- Disagree
- Somewhat disagree
- Somewhat agree
- Agree
- Strongly agree

The automated or [good model note] case feedback motivated me to improve my patient notes.

- Strongly disagree
- Disagree
- Somewhat disagree
- Somewhat agree
- Agree
- Strongly agree

The automated or [good model note] case feedback will help prepare me for future simulated patient case-based examinations.

- Strongly disagree
- Disagree
- Somewhat disagree
- Somewhat agree
- Agree
- Strongly agree

The case feedback changed my perception of my performance.

- Strongly disagree
- Disagree
- Somewhat disagree
- Somewhat agree
- Agree
- Strongly agree

Feedback from the automated grading system or [good model note] will help me develop history gathering skills.

- Strongly disagree
- Disagree
- Somewhat disagree
- Somewhat agree
- Agree
- Strongly agree

Feedback from the automated grading system or [good model note] will help me focus my physical examination on relevant areas.

- Strongly disagree
- Disagree
- Somewhat disagree
- Somewhat agree
- Agree
- Strongly agree

Feedback from the automated grading system or [good model note] will help me learn differential diagnosis.

- Strongly disagree
- Disagree
- Somewhat disagree
- Somewhat agree
- Agree
- Strongly agree

Feedback from the automated grading system or [good model note] will help me learn about diagnostic testing choices.

- Strongly disagree
- Disagree
- Somewhat disagree
- Somewhat agree
- Agree
- Strongly agree

The automated note grading system accurately scored my answers.

- Strongly disagree
- Disagree
- Somewhat disagree
- Somewhat agree
- Agree
- Strongly agree

The standard my answer was graded against was clear.

- Strongly disagree
- Disagree
- Somewhat disagree
- Somewhat agree
- Agree
- Strongly agree

It was clear why a patient note grading item was achieved or missed.

- Strongly disagree
- Disagree
- Somewhat disagree
- Somewhat agree
- Agree
- Strongly agree

My ability to write a patient note for this chief complaint BEFORE and AFTER the simulation case feedback:

|  | 0% (Not at all Capable) (1) | 10% (2) | 20% (3) | 30% (4) | 40% (5) | 50% (Somewhat Capable) (6) | 60% (7) | 70% (8) | 80% (9) | 90% (10) | 100% (Completely Capable) (11) |
| --- | --- | --- | --- | --- | --- | --- | --- | --- | --- | --- | --- |
| BEFORE (1) |  |  |  |  |  |  |  |  |  |  |  |
| AFTER (2) |  |  |  |  |  |  |  |  |  |  |  |

How would you improve the automated feedback on your patient note?

[Open text response]
